# Changes in consumer dynamics on general e-commerce platforms during the COVID-19 pandemic: An exploratory study of the Japanese market

**DOI:** 10.1016/j.heliyon.2022.e08867

**Published:** 2022-02-02

**Authors:** Yuki Inoue, Masataka Hashimoto

**Affiliations:** a Graduate School of Humanities and Social Sciences, Hiroshima University, Higashi-senda 1-1-89, Naka-ku, Hiroshima-shi, 730-0053, Japan; b Graduate School of Global Business, Meiji University, 1-1, Surugadai, Kanda, Chiyoda-ku, Tokyo, 101-8301, Japan

**Keywords:** Platform-based market, E-commerce, COVID-19, Platform pricing, Product diversity, Delivery services, Consumer dynamics

## Abstract

This study analyzed the changes in consumers' use dynamics of general e-commerce (EC) platforms (e.g., Amazon.com) during the coronavirus disease 2019 (COVID-19) pandemic. We initially supposed that the significance of consumer benefits, including pricing, product variety, and delivery services, on the platforms would decrease and the value of using an EC platform itself would increase due to the pandemic, based on which we conducted a comparative analysis of questionnaire data from 2,119 Japanese consumers who use general EC platforms. The data were obtained in November 2018 and January 2021. Our analysis has two parts, the first designed as a conjoint analysis to statistically analyze the changes in consumers' sense of values for pricing, variety of goods, stability and quality of delivery services, and basic benefits of using EC platforms; and the second part designed to statistically analyze the changes in comprehensive items when using EC platforms. We categorized the dataset into men and women and further clustered them based on the patterns of consumers' sense of factors when using EC platform. The analysis results were inconsistent with our initial supposition, in that a non-negligible proportion of consumer clusters showed an increase in the significance of factors, including pricing, product variety, and delivery service, and a decrease in the basic benefits of using EC platforms. Regarding the results of the analysis of comprehensive items, the only commonly observed change for most clusters of both men and women was an increase in the use of package drop. The results indicate that changes in consumers’ sense of using EC platforms due to the pandemic were not as simple as supposed because the pandemic caused various changes in the need mechanism of consumers of EC platforms.

## 1 Introduction

The market size of e-commerce (EC) platforms, such as Amazon.com, is increasingly expanding. Notably, the coronavirus disease 2019 (COVID-19) pandemic forced people to stay at home, highlighting the significance and usefulness of EC platforms (Cavallo et al., 2020). Although measures against COVID-19 differ across countries, most countries have prohibited or requested citizens to reduce going outside (Sawangchai et al., 2020). Even where governments did not restrict people from going out, people intentionally avoided physical contact with others in their daily lives (Civelek et al., 2021). In these circumstances, people are now used to shopping via EC platforms rather than going out.

Some studies have suggested that COVID-19 has directly and indirectly contributed to the diffusion of EC platforms, facilitating shifts in the format of revenue streams of many businesses from offline to online (Barnes, 2020). More retailers have explored EC and shifted to EC businesses due to the pandemic (Beckers et al., 2021). The pandemic resulted in the necessity to strategically design EC platforms for businesses to ensure the continuous consumption behaviors of customers (Tran, 2021). As an aspect of technological diffusion, the COVID-19 pandemic has expanded the diffusion of online shopping, including EC platforms (Kim, 2020). Additionally, it can be considered a diffusion mechanism of innovation owing to the crisis (Dannenberg et al., 2020). Specifically, Hashem (2020) suggested that women are more affected by the pandemic than men. From a geographical perspective, researchers report that people living in large cities have a decreased risk perception of online purchases during the pandemic (Gao et al., 2020). Other researchers have focused on the significant expansion of food delivery services in EC-platform-based markets (Chang and Meyerhoefer, 2021; Muangmee et al., 2021). Thus, the COVID-19 pandemic has facilitated the digital transformation of both businesses and consumers through expansion of the use of EC technologies. Therefore, the influence of COVID-19 with respect to EC platforms is regarded as a new subject in EC research (Kumar et al., 2021).

This study investigated the positive aspects of COVID-19 in the diffusion of EC platforms. Specifically, we focused on consumers’ views of EC platforms and examined the changes in their dynamics of EC platform use before and after the onset of the pandemic. The process of purchases on general EC platforms, such as Amazon.com, typically consists of the following: (a) Consumers select products on the web platform and purchase them through the platform, (b) the platform or exhibiters request delivery to delivery firms, and (c) delivery firms deliver the purchased products from the platform firm or exhibiters to consumers (Inoue et al., 2019). In this context, fees for using the platform, available product variety on the platform, and delivery services assigned through the platform are significant factors for consumers on general EC platforms (Inoue et al., 2019). To the best of our knowledge, previous studies have not systematically analyzed how the significance of these factors in platform-based markets has changed due to the pandemic.

As stated above, the crisis of this pandemic can be considered a diffusion mechanism of innovation (Dannenberg et al., 2020). Many people prefer to stay at home and use EC platforms despite the fact that the performance of the platforms is consistent with that before the pandemic. Therefore, we supposed that the significance of consumers' benefits, such as pricing, product variety, and delivery services, on the platforms could decrease, and the value of using EC platforms could increase due to the pandemic. Consequently, our research question was: “*Due to the COVID-19 pandemic, has the significance of factors, including pricing, product variety, and delivery services, decreased for consumers? Meanwhile, did the value of using EC platforms increase?*” The answers to this question would be valuable for practitioners and researchers who focus on EC platforms. For practitioners, if the answer to this question is “Yes,” they ought to seek new differentiation factors for their platforms. However, even if the answer is “No,” if we observe any changes in consumers' perceptions of pricing, product variety, delivery services, and value of platform use, they might need modification to serve as differentiation points of their platforms. Similarly, unless we never observe any changes in these factors owing to the pandemic, such results might suggest new avenues of research for platform researchers. Thus, to answer this research question, this study analyzes the implications of the changes in consumers’ EC platform use dynamics during the pandemic.

To this end, a comparison analysis was conducted between the data of consumers' platform use before and during the pandemic. Regarding the focus of this study, we adopted an exploratory rather than a confirmatory (or hypothetical) study. We utilized a questionnaire survey conducted in November 2018 of consumers of Japanese EC platforms and carried out a survey with the same content in January 2021. Although these surveys were conducted in different months, we addressed the potential bias caused by seasonality. Then, we statistically compared the data collected. The results of this study confirmed some changes in consumers’ dynamics of EC platform use and suggest the implications of these changes.

## 2 Literature review

We first explain “indirect network effects,” which are fundamental evolutionary mechanisms of platform-based markets, and then a review three factors examined in this study: pricing, variety of goods, and stability and quality of delivery services in EC platforms.

### 2.1 Indirect network effects

Some types of platforms, including EC platforms, serve as intermediaries between two or more groups and form two-sided (or multi-sided) markets (Gawer, 2014; Gawer and Cusumano, 2014; Thomas et al., 2014). In these types of markets, the indirect network effect implies that a relationship exists wherein the scale of actors on one side connects to the expected benefits of actors on the other side through participation on the platform, and this relationship is mutually consistent (Armstrong, 2006; Evans, 2003; Hagiu and Wright, 2015; Rochet and Tirole, 2003, 2006). For example, the expected benefits of consumers on typical EC platforms would increase as product variation and number of sellers increase because consumers expect to find preferable options on these platforms and thus gain more satisfaction. Conversely, the expected benefits of sellers would increase as the number of consumers increases because sellers expect their products/services to be purchased more frequently. Through such mutually reinforcing dynamics, the indirect network effect can cause exponential market growth (Nair et al., 2004). This nature theoretically connects to the “winner-take-all” situation of competition among platforms (Eisenmann, 2007; Eisenmann et al., 2006; Frank and Cook, 1995). Therefore, platform owners are likely eager to acquire more installed bases (consumer size) and greater availability of goods (Clements and Ohashi, 2005; Schilling, 2002; Zhu and Iansiti, 2012). In fact, the strategy of platform owners to achieve this is effective in terms of competition (Inoue and Tsujimoto, 2018). However, despite the success of platform owners in acquiring more actors on both sides, the persistence of the market could be lost if actors on either side are unable to stay in the market (Inoue and Tsujimoto, 2018). Thus, platform owners should promote a virtuous cycle of indirect network effects and maintain them by ensuring continuous expected benefits on the corresponding platforms. As explained previously, on EC platforms, three major factors, namely, pricing, variety of goods and needs, and stability and quality of delivery services, are regarded as significantly related to consumers’ benefits (Inoue et al., 2019), and thus are significant in creating and maintaining indirect network effects for consumers in EC platform-based markets.

### 2.2 Pricing

“Pricing” refers to how platform owners set the platform use fee. One study suggests that pricing of platforms should be set based on the strength of indirect network effects in two-sided (or multi-sided) markets (Yoo et al., 2002). Rochet and Tirole (2003) suggest that pricing can be set depending on the demand elasticity between sides by following the Ramsey rule. For platform types such as an EC platform, the standard pricing scheme is either fixed fees, such as membership fees, or variable fees, such as transaction fees (Hagiu, 2006). To acquire a maximum market share, fees can be set in a negative direction for one (or the other) side by providing incentives or subsidies (Caillaud and Jullien, 2003; Lin et al., 2011). Moreover, actors’ participation status on multiple platforms (called multihoming) can be considered in pricing settings (Armstrong, 2006). Thus, appropriate (or suboptimal) pricing can vary depending on the relationship between actors and market structure. In an actual market, EC platforms with suboptimal pricing schemes will either not grow or be naturally eliminated from competition (Inoue et al., 2019b).

### 2.3 Variety of goods

A platform-based market can have a collection of various goods provided by sellers with different types of management resources, as well as various needs demanded by buyers with diversified profiles (Inoue, 2021). Such a variety on a platform is crucial for continuous benefit from the indirect network effect. For buyers (i.e., consumers on EC platforms), a variety of goods is necessary to encourage more purchases and is associated with their perceived value of the respective platform (Kim and Ammeter, 2018). Sellers (i.e., exhibitors in EC platforms) cannot obtain sufficient benefits on the platform if the need for their products does not arise (Inoue and Tsujimoto, 2018). Unlike the supply chain, platform-based markets typically allow sellers' autonomy (Jacobides et al., 2018). Although platform owners find it difficult to control the market owing to this autonomy, it affords buyers the opportunity to receive more innovative goods provided by sellers (Inoue, 2021). However, because the variety of goods in a platform-based market easily exceeds the limitation of consumers’ perception, preparation of appropriate search and recommendation systems is significant to convert the variety of goods into a capturable value on the platforms (Brynjolfsson et al., 2011).

### 2.4 Stability and quality of delivery services

As “delivery” is a crucial part of basic business model of EC platforms, such as Amazon, the stability and quality of delivery services are significant factors in the growth and continuity of EC platform-based markets (Inoue et al., 2019). An example of “stability of delivery service” is delivery times without delays, whereas an example of “quality of delivery service” is short delivery times (Inoue et al., 2019). Naturally, the concern with delivery services is not restricted to these examples. The content of delivery services can be associated with consumers’ satisfaction (Chen et al., 2011; Vasić et al., 2021). Specifically, delivery plays a role in mediating the effect of customer experience of an EC platform (website or application) on consumer satisfaction (Vakulenko et al., 2019). One study further indicated that factors such as convenience, communication, reliability, and responsiveness are crucial in achieving higher satisfaction levels (Hong et al., 2019). Consumers and sellers (or exhibitors) derive satisfaction from superior delivery services on platform-based markets (Yu et al., 2015). Given the significance of delivery services, a previous study suggested that compensation by price adjustment becomes rational if a deterioration of delivery services is inevitable during, for example, busy seasons (Zhang et al., 2016). However, if platform owners disregard or squeeze delivery service providers through the consideration of price adjustment, they could risk a large-scale withdrawal of delivery firms, which might even lead to a collapse of the market (Inoue et al., 2019). Therefore, platform owners must optimize the benefits of consumers, sellers, and delivery firms to maintain the stability and quality of delivery service.

## 3 Materials and methods

Consistent with the proposed research question, we analyzed how consumers' sense of the significance of factors including pricing, product variety, delivery services, and value of using general EC platforms was changed by the COVID-19 pandemic. We conducted a questionnaire survey of consumers of general EC platforms in November 2018 and January 2021. Prior to the start of this study, we had conducted a questionnaire survey in 2018 to analyze how consumers on EC platforms consider the stability and quality of delivery services in comparison with pricing and availability of variety of goods. Understandably, because we did not expect the COVID-19 pandemic, the 2018 questionnaire did not include items that directly asked about the influence of the pandemic. In 2021, we had two options on how to conduct the questionnaire survey for this study: (a) designing a new questionnaire to capture the changes in consumers’ sense of factors on EC platforms, and (b) using the same questionnaire sheet as in 2018 to analyze the response changes between 2018 and 2021. The advantage of the first option is that it can directly capture changes from the respondents, while the disadvantage is that the captured change would include greater subjectiveness than the second option. Additionally, analysis of data obtained from the first option could not have a reference point for changes, whereas with the second option, the 2018 data could serve as the reference point. Accordingly, we selected the second option and intentionally set all structures and contents of the survey in 2021 the same as in 2018 to allow statistical comparisons between the 2018 and 2021 datasets.

The details of the questionnaire are presented in Appendix A. Due to the differences in the research purpose between this study and our previous study in 2018, some questions were not used in the analysis of this study. However, to eliminate potential bias, we asked all the same questions in 2021 as in the 2018 survey. Although the survey months were different in 2018 and 2021 (the former was in November and the latter in January), we originally asked the respondents to answer the questions based on their experience of EC platform use in the past six months to mitigate bias caused by seasonality under the assumption that this treatment could absorb the bias due to the months when the survey was conducted. Furthermore, regarding questions potentially influenced by seasonality (specifically, the question “Frequently purchased goods”), we searched for other supportive evidence that the results are not caused by seasonality (presented in the latter part of Section 4.1.2). Thus, this study captured data in November 2018 and January 2021 with a similarly designed survey and statistically analyzed the changes in answers before and after the onset of the pandemic.

### 3.1 Sampling

We prepared a questionnaire and distributed it via the Internet through Macromill, Inc. This firm is one of the largest investigation firms in Japan and has approximately 10 million respondents prepared to answer Internet surveys. Other than the condition “adult of 20 years old or older,” the sampling method was random. The respondents were given incentives to earnestly answer the questionnaires in the form of electronic points that could be exchanged for goods or money if the answer was valid.

We considered that those who rarely use EC platforms or use them only for business were not appropriate for the survey. Hence, to exclude such respondents, we set two questions for screening: (SQ1) How frequently do you use EC platforms? (SQ2) What are the types of products you purchase on EC platforms? If the respondent answered “more than once a month” for SQ1 and “products for personal and daily goods” for SQ2, they were directed to the main question items (described in the next section). Here, the survey explained that the EC platforms in this questionnaire were restricted to a platform type with associated delivery services and providing various products, such as Amazon.com. Only respondents who passed these screenings could move to answer the full questionnaire. The full questionnaire is provided in Appendix A. We asked Macromill, Inc. to collect 2,000 samples in the 2018 survey and 1,000 samples in the 2021 survey, and they acquired 2,060 samples in 2018 and 1,030 samples in 2021. Here, the restriction of the research budget resulted in a difference in sample size between 2018 and 2021. However, because we did not use any statistical methods that assume the same sample size for comparison targets, we considered this difference acceptable.

After data collection, we performed further screening to extract samples with strongly reliable answers. This aims to prevent occurrence of clusters that show logically wrong mechanisms of platform use by cumulating logically strange answers in the analysis. The details of this method are explained in Section 3.2. After this additional screening, we identified 1,423 and 696 valid samples for 2018 and 2021, respectively, for use in the analysis. Although we removed approximately 30% of the samples in the dataset, this study prioritized the high reliability of responses and the final calculated results.

Regarding the screened samples, the breakdown of the samples in 2018 was 657 men and 766 women, with an average age of 46.12 during the survey period. For 2021, 380 men and 316 women were sampled, with an average age of 48.63 years. Although we confirmed a bias in the proportion of sex between the two surveys, it was not significant because this study separated the samples in the analysis by sex. Furthermore, although the average age between the two surveys slightly varied, we regarded it as an expansion of EC platform consumers.

### 3.2 Questionnaire items and analytical approaches

The questionnaire was divided into two parts. The first part was designed for the conjoint analysis of consumers' sense of values for the three factors described in Section 2.2 (pricing, variety of goods, and stability and quality of delivery services). The second part asked comprehensive questions to capture consumers’ usage patterns of EC platforms. “Age” and “Job” were captured from the registered information at the survey monitor, and not from this specific questionnaire. The major part of this study is the first part, whereas the second part is complementary to the results of the first part. However, in the questionnaire sheet, questions of the second part were presented first because their answers were easier (see Appendix A).

In the first part, a conjoint analysis of consumers’ sense of values for the three factors described in Section 2.2 was conducted. We separately addressed two factors regarding delivery services: stability and quality, and therefore considered four factors. We set a hypothetical situation and asked the respondents, “Please imagine a situation where you buy products that are priced in general retail stores at 5,000 Japanese yen (approximately USD 45–50). If you are presented the following conditions for a platform, will you use this platform? Or will you use a retail store?” Then, we proposed a series of conditions presented as combinations of four factors, with five scoring levels for each, as follows:

∗ **Pricing:** {1,000 yen discount, 500 yen discount, the same price as in a retail store, 500 yen more expensive, 1,000 yen more expensive}.
∗ **Variety of goods:** {1/4 of the store, 1/2 of the store, same level as the store, double the store, four times the store}.
∗ **Probability of delay in delivery (stability of delivery services):** {0%, 25%, 50%, 75%, 100%}.
∗ **Minimum delivery period (quality of delivery services):** {0 days, 3 days, 6 days, 9 days, 12 days}.

By using an orthogonal array, the combination of levels was decreased to 25 condition patterns, and the order of the 25 condition patterns was randomized.

The captured data from this part were analyzed using logistic regression. The dependent variable is a binary variable defined as the use of an EC platform with the condition presented = 1, otherwise = 0. The explanatory variables are based on these four factors. Here, we used the natural logarithms of the variables of the variety of goods to convert them to equal-interval variables, similar to the other three factors. We then reversed the order of the three factors—pricing, probability of delay delivery, and minimum delivery period—to ensure that a higher value implies a positive experience for consumers. Finally, we standardized the values of each variable as the *Z*-score (mean = 0, *SD* = 1) and set them as explanatory variables. In summary, the variables in the logistic regression analysis are defined as follows:

∗ **Dependent variable:** Intention of platform use in the proposed condition (Yes = 1, No = 0).
∗ **Explanatory variables:** (a) Better pricing; (b) Greater variety of goods; (c) Fewer delivery delays; (d) Faster delivery.

We confirmed that some of the samples were inadequate for analysis and thus removed them using the following steps. As described in Section 3.1, this removal aims to prevent occurrence as clusters, which would indicate logically wrong mechanisms about platform use through the cumulation of logically strange answers. First, some respondents answered that all the values of the dependent variables were either 0 or 1. Therefore, we omitted the samples with a standard deviation of zero on the dependent variables. Second, we omitted those for which the relationship between the dependent variable and explanatory variables, according to the answers of some of the respondents, was logically incorrect (e.g., an answer indicating that platform use probability increases as delay rate becomes higher). To exclude these samples, we calculated the Spearman's rank correlation coefficient between the dependent variable and each explanatory variable for each sample and omitted the samples with a coefficient value below −0.1. This process corresponds to “additional screening” described in Section 3.1.

Table 1 summarizes the questions in the second part. In this table, the categories of questions are presented on the left side, and question items and options or input in each question category are provided on the right side. In the statistical analysis, the answers were compared using Fisher's exact test or the Wilcoxon signed-rank test. The former was adopted when a question required a binary answer (Yes or No; Applicable or Not applicable), and the latter was adopted in other cases.

**Table 1:** Comprehensive questions regarding the use patterns of EC platforms.

Category	Items
Age	Input the numerical value.
Job	Select the most applicable: {1. Company employee; 2. Public worker; 3. Company executive; 4. Self-employed worker; 5. Freelancer; 6. Housewife/househusband; 7. Part-time worker; 8. Student; 9. Other occupations; 10. No occupation}.
Times available to receive delivered goods	Select all available times from a combination of days of the week and time slots. Days of the week: {Monday, Tuesday, Wednesday, Thursday, Friday, Saturday, Sunday}. Time slots: {Morning: 9 am–noon; Afternoon: noon–3 pm; Dusk: 3 pm–6 pm; Night: 6 pm–9 pm}.
Way of receiving goods	Write the percentage for the methods of receiving goods for {1. Self at home; 2. Self at workplace; 3. Family or friends; 4. Delivery locker; 5. Interim storage services at convenience stores or delivery firms and then collect; 6. Use of package drop (known also as unattended delivery) services; 7. Others}
Frequently purchased goods	Select all frequently purchased goods: {1. Book or journal; 2. DVD, music CD, video game, or hardware/software; 3. Consumer electronics; 4. Computer-related supplies; 5. Household utensils; 6. Food or drink; 7. Medicine or cosmetics; 8. Children's products or toys; 9. Clothing or accessories; 10. Sporting or outdoor; 11. Motor vehicle related; 12. Industrial machinery or R&D related; 13. Others}. Here, this categorization was defined by referring to the categories found on website of Amazon.co.jp in November 2018.
Dissatisfaction factors	Select all frequently experienced dissatisfaction factors: {1. Expensive; 2. Low variety of goods; 3. Excessive packaging; 4. Delay in delivery; 5. Rough treatment of parcel; 6. Bad attitude of the delivery person; 7. Degradation of perishables; 8. Others}.
Paid membership	Select all current paid-up memberships: {1. Amazon Prime; 2. Rakuten Premium; 3. Yahoo! Premium; 4. Others; 5. No paid membership}. Here, if a respondent selected Option 5, they could not select other options.

### 3.3 Procedure of the statistical analysis

The analysis conducted in this study was divided into two parts: Part A (i.e., basic analysis) and Part B (i.e., a more detailed analysis).

In Part A, the questionnaire data of 2018 and 2021 for men and women were statistically compared. In the first part of the analysis, we set the interaction terms between explanatory variables and 2021 dummy variables and then analyzed the changes in consumers' sense of value of the four explanatory variables and basic perceived value of the use of EC platforms. In the second part of the analysis, statistical significance tests, including Fisher's exact test and the Wilcoxon signed-rank test, were conducted.

In Part B, to analyze consumers’ patterns for platform use mechanisms in more detail, the dataset was clustered by similarity in the sense of value for the four explanatory variables from both the 2018 and 2021 data and on men and women separately. Thereafter, we statistically compared the clustered dataset in the same manner as in part A. The detail of the procedure is as follows:

(1) For the 2018 data, we calculated the Spearman's rank correlation coefficient for each sample between the dependent variable (i.e., binary variables about intention of platform use in the proposed condition) and each explanatory variable (i.e., five-level interval scale variables about pricing, variety of goods, probability of delay in delivery, and minimum delivery period). Thus, each sample had four values for the clustering calculation. We then prepared a matrix in which the four correlation coefficient values were aligned horizontally and the values in each sample were aligned vertically. This matrix was prepared separately for men and women.
(2) For each matrix of men and women generated by procedure (1), we created a dendrogram using Ward's method.
(3) By observing the generated dendrogram, we divided the samples into an appropriate number of clusters. Thus, we set four clusters for men and five clusters for women.(4) Similarly, we implemented procedures (1), (2), and (3) for the 2021 data.
(5) For each clustered sample group, we conducted a logistic regression analysis using the method explained in Section 3.2. Therefore, we identified patterns for each cluster on how the four explanatory variables influence a dependent variable.
(6) By observing the dendrogram figures and confirming the calculated results of logistic regression analysis for each cluster, we associated clusters of 2018 with those of 2021.
(7) For each combination of associated clusters, we conducted a statistical analysis in the same manner as in Part A. Here, we confirmed that no multicollinearity exists for all logistic regression analyses (maximum value of VIF was 2.75, which was below the cutoff value of 10).

## 4 Results

### 4.1 Analysis of part A: statistical results for the entire dataset

#### 4.1.1 Results of the changes in the sense of value for the four factors on platform use

Table 2 presents the results of the changes in the sense of value for the four explanatory variables and of the basic value of platform use for the entire dataset through a logistic regression analysis. At this analytical level, changes were not observed for men, while a decrease for the “Minimum delivery period” and an increase in the basic value of use (i.e., 2021 dummy) showing statistical significance (each *p* < 0.05, 0.01) were observed for women. Thus, the results for the entire dataset indicate obvious changes in women than those in men.

**Table 2:** Change in the sense of value for four explanatory variables on platform use on the entire dataset.

	Men			Women		
	Coef.	SD	*p*-value	Coef.	SD	*p*-value
Better pricing	1.840	0.028	0.000	1.953	0.028	0.000
Greater variety of goods	0.251	0.022	0.000	0.341	0.021	0.000
Fewer delivery delays	0.333	0.022	0.000	0.461	0.022	0.000
Faster delivery	0.561	0.022	0.000	0.657	0.021	0.000
Better pricing × 2021 dummy	−0.002	0.047	n.s.	−0.049	0.051	n.s.
Greater variety of goods × 2021 dummy	−0.037	0.036	n.s.	−0.051	0.038	n.s.
Fewer delivery delays × 2021 dummy	0.041	0.037	n.s.	−0.069	0.039	0.081
Faster delivery × 2021 dummy	0.004	0.036	n.s.	−0.086	0.038	0.025
2021 dummy	−0.051	0.037	0.172	0.137	0.040	0.001
Constant	−0.662	0.022	0.000	−0.925	0.022	0.000
Pseudo *R*-squared	0.522			0.545		

Note: To improve readability, cells with *p-*values higher than 0.1 are denoted by “n.s.” (not significant).

#### 4.1.2 Results for changes in consumers’ comprehensive items on EC platforms

Table 3 present the results for the changes in consumers’ comprehensive items on EC platforms. In this table, the percentage values that respondents answered for each question item or the average values of input are provided for each combination of year and sex. The adjacent cells present the *p*-values calculated through the statistical analysis of the differences between the 2018 and 2021 values. For better readability, this table only shows the results of question items that showed *p-*values of less than 0.1 for either men or women. However, because this table still contains many items, we only discuss those with statistical significance (i.e., *p* < 0.05) in the main text.

**Table 3:**
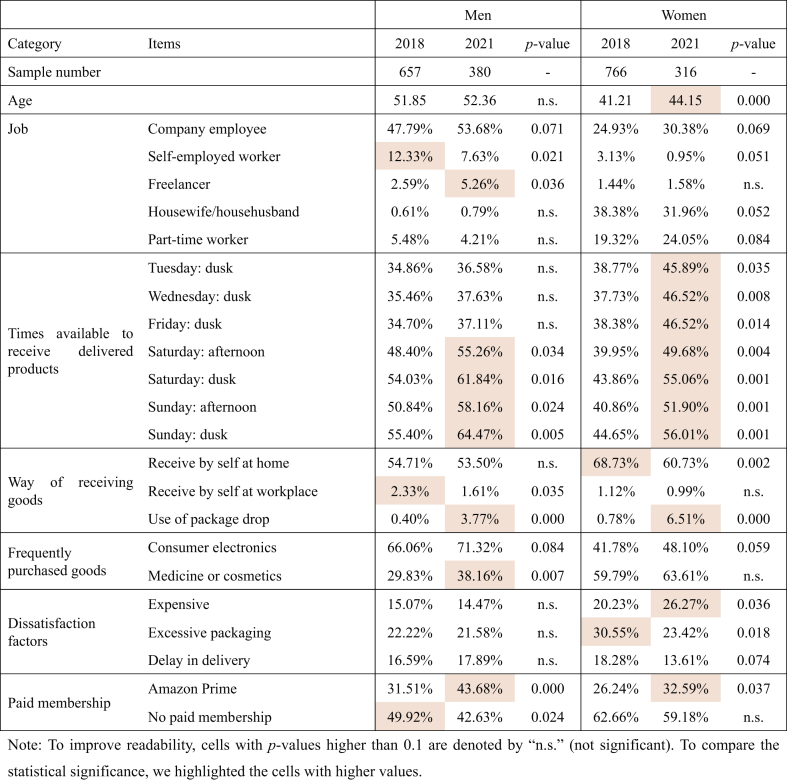
Change in consumers’ comprehensive items on EC platforms on the entire dataset.

Regarding changes in “profile,” a statistically significant decrease was observed in the number of self-employed workers and an increase in freelancers for men (both *p* < 0.05). This implies a change in the work environment due to the pandemic. In the case of women, the average age of the respondents significantly increased by about 3 years (*p* < 0.01), implying an expansion in the number of users for EC platforms. Regarding changes to “Available times to receive delivered products,” availability in the afternoon and dusk on Saturday and Sunday significantly increased for both men and women (*p* < 0.05, *p* < 0.01). Additionally, availability at dusk on Tuesday, Wednesday, and Friday showed a significant increase for women (*p* < 0.05, *p* < 0.01). These results imply not only the influence of the restrictions of going out but also an increase in the acceptability of new delivery styles for consumers. Regarding changes in “Frequently purchased goods,” only the purchase of medicine or cosmetics by men showed a significant increase (*p* < 0.01). Thus, despite the change being small, we confirmed a change in the purchase of goods. For change in “Dissatisfaction factors,” a statistically significant increase was observed in dissatisfaction in the expensive category and a decrease in excessive packaging (both *p* < 0.05) for women, but not for men. Thus, changes in these attitudes differed between men and women. Finally, for change in “Paid membership,” a statistically significant increase for Amazon Prime was observed for both men and women (*p* < 0.05, or *p* < 0.01). Additionally, the proportion of men who did not have any paid membership significantly decreased (*p* < 0.05). Thus, paid membership acceptance of consumers on EC platforms has improved.

As described previously, we confirmed the influence of seasonality on the results of the question items of “Frequently purchased goods.” The results indicate that consumer electronics, medicine, and cosmetics showed marginally significant increases or statistically significant increases (*p* < 0.10 or 0.05, respectively). As another source, we found supportive results from a survey report conducted by the Ministry of Economy, Trade and Industry (METI) in Japan (METI, 2021). The report shows that the market size of consumer electronics in EC increased by 28.79%, and increased by 17.79% for medicine or cosmetics from 2019 to 2020 in Japan. This report did not directly show an increase in the individual rate of frequently purchased goods, as in our study. However, this finding may indicate that the increase observed in our study was not due to seasonality. Moreover, the METI report showed a large increase in market size for other product categories for which our study did not show a statistically significant increase. We considered that an increase in these product categories could be absorbed by other specialized platforms, because our survey mainly focused on platforms dealing with comprehensive products, such as Amazon. For example, an increase in books may be absorbed by Kindle, that of apparel may be absorbed by ZOZOTOWN (the largest apparel EC platform in Japan) and other such platforms, and that of foods would be absorbed by Uber Eats and other such platforms. These points remain as limitations and are discussed in the section on limitations and future works.

### 4.2 Analysis of part B: statistical results on clustered data

#### 4.2.1 Results of changes in the sense of value of the four factors on platform use

Table 4 presents the results of changes in the sense of value of the four explanatory variables on platform use in the clustered dataset by logistic regression analysis. First, we examine the results for men. Cluster 1 focused on better pricing in the 2018 data. In this cluster, the basic value of using the EC platform (i.e., expressed as 2021 dummy) had significantly increased by 2021 (*p* < 0.01). Cluster 2 is a group with neither high nor low focus for any of the four factors in the 2018 data. In this cluster, the focus on better pricing had significantly increased by 2021 (*p* < 0.01). Cluster 3 focused on faster delivery in the 2018 data. In this cluster, the focus on faster delivery had further significantly increased by 2021 (*p* < 0.01). However, the basic value of using the EC platform had significantly decreased (*p* < 0.01). Cluster 4 focused on greater product variety and fewer delays in delivery in the 2018 data. In this cluster, the focus on better pricing had significantly increased by 2021 (*p* < 0.05). However, the focus on greater product variety and the basic value of using the EC platform had significantly decreased (both *p* < 0.05). Thus, although the analysis of all the data presented in Table 2 did not exhibit a change due to the pandemic, analysis of the clustered data (Table 4) showed some change in the sense of value for the four factors on platform use among men.

**Table 4:**
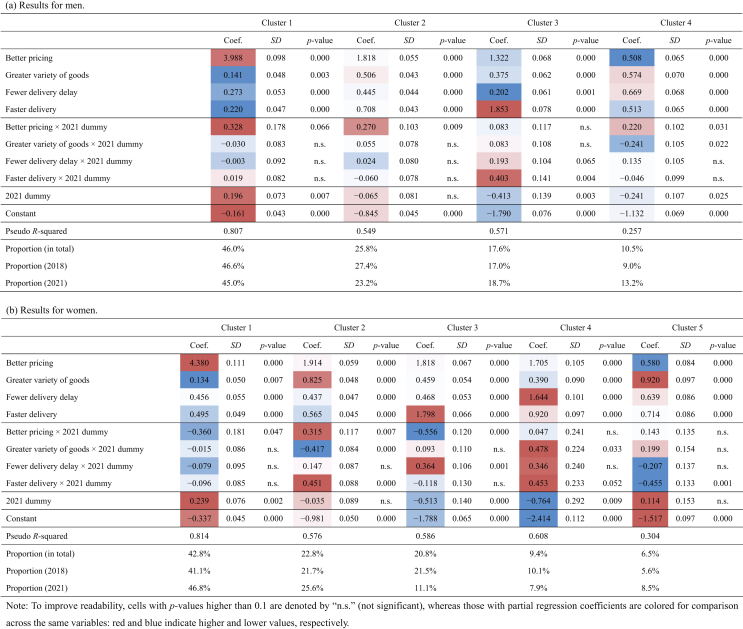
Changes in the sense of value for four explanatory variables on platform use on the clustered dataset.

Next, we examine the results for women. Similar to men, Cluster 1 focused on better pricing in the 2018 data. In this cluster, the focus on better pricing significantly decreased (*p* < 0.05). However, the basic value of using the EC platform had significantly increased by 2021 (*p* < 0.01). Cluster 2 focused more on product variety in the 2018 data. This cluster's focus on better pricing and faster delivery significantly increased by 2021 (both *p* < 0.01), but the focus on greater product variety had significantly decreased by 2021 (*p* < 0.01). Cluster 3 focused on faster delivery in the 2018 data, similar to Cluster 3 of men. In this cluster, the focus on fewer delays in delivery significantly increased by 2021 (*p* < 0.01). However, the focus on better pricing and the basic value of using an EC platform had significantly decreased (both *p* < 0.01). Cluster 4 focused on fewer delivery delays in the 2018 data. In this cluster, the focus on a greater variety of goods had significantly increased by 2021 (*p* < 0.05), but the basic value of using an EC platform had significantly decreased (*p* < 0.01). Finally, Cluster 5 focused on a greater variety of goods in the 2018 data, similar to Cluster 2, but focused less on better pricing and more on delivery services. In this cluster, the focus on faster delivery had significantly decreased by 2021 (*p* < 0.01). Thus, we found that patterns of change due to the pandemic largely differed among women from those of men.

#### 4.2.2 Results of changes in consumers’ comprehensive items on EC platforms

Table 5 shows the results of the changes in the comprehensive items of consumer behavior on EC platforms in a clustered dataset. Following the structure of Table 3, the percentage values that respondents answered suitably for each question item or the average values of input are presented for each combination of year and sex. The adjacent values are the *p*-values calculated by statistical analysis of the differences between the 2018 and 2021 values. To improve readability, we separated the tables for men and women, and only the items in this table are presented in Table 3.

**Table 5:**
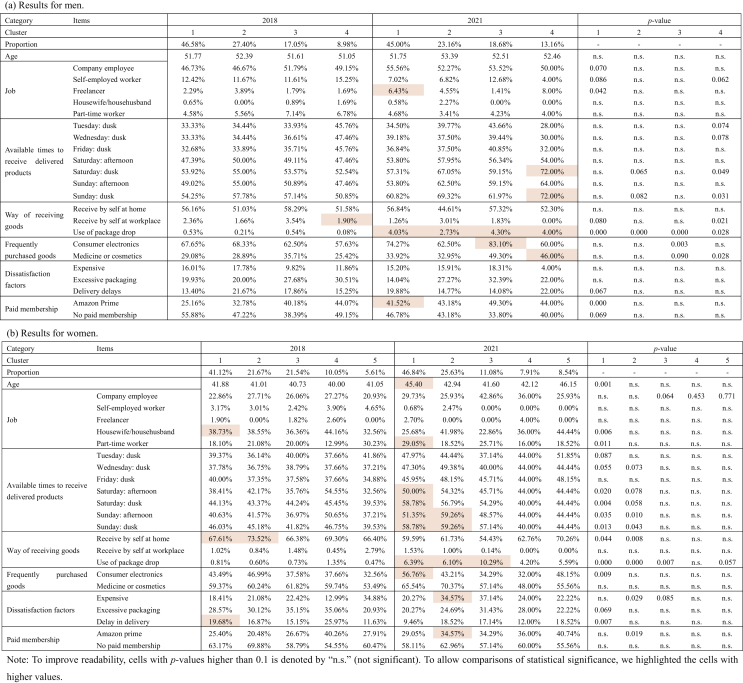
Changes in consumers’ comprehensive items on EC platforms in the clustered dataset.

First, we explain the results for men. A confirmed significant change common across all clusters was an increase in the use of package drops (*p* < 0.01 or *p* < 0.05). Changes in other items were observed differently in each cluster. Regarding the change in Cluster 1, there was an increase in freelancers (approximately 4%) and an increase in paid membership for Amazon Prime (approximately 16%) that were statistically significant (*p* < 0.05, *p* < 0.01). Cluster 2 did not show an additional statistically significant change, although changes in some items showed marginal significance. For Cluster 3, the selection of consumer electronics as frequently purchased goods increased by approximately 20%, and this was statistically significant (*p* < 0.01). In Cluster 4, an increase in the availability of goods was found at dusk on Saturday and Sunday (approximately 20% increase for both). A decrease was observed in receive by self at workplace (0%), and a statistically significant increase in purchase of medicine or cosmetics showed an approximately 20% increase. Thus, we confirmed the different changes caused during the pandemic across the four clusters of men.

Turning to women, similar to the results for men, we found a significant increase in the use of package drops for most clusters (all *p* < 0.01). However, the change in Cluster 5 was marginally significant (*p* < 0.10), and that in Cluster 4 was not significant. Changes in other items were observed differently in each cluster, similar to the case for men. Regarding the changes in Cluster 1, the following exhibited a statistical significance (*p* < 0.05 or *p* < 0.01): an increase in age (approximately 3.5 years), decrease in number of housewives (approximately 13%), an increase in number of part-time workers (approximately 11%), and availability for receiving in the afternoon and dusk on Saturdays and Sundays (from 10% to 15%), a decrease in receive by self at home (around 8%), an increase in purchase of consumer electronics (approximately 13%), and a decrease in dissatisfaction for delay in delivery (10%). Cluster 2 showed an increase in the availability to receive in the afternoon and dusk on Sundays (approximately 14%–18%), a decrease in receive by self at home (approximately 12%), an increase in dissatisfaction for high costs on the platform (an increase of 13%), and an increase in paid membership for Amazon Prime (approximately 14%), was statistically significant (*p* < 0.05 or 0.01). For Clusters 3, 4, and 5, no statistically significant difference was observed. Thus, similar to the results for men, we confirmed different changes across the five clusters of women due to the pandemic.

## 5 Discussion

This study established the research question, “*Due to the COVID-19 pandemic, did the significance of factors, including pricing, product variety, and delivery services decrease for consumers? Meanwhile, did the value of using EC platforms increase?*” We then conducted a comparative analysis between November 2018 and January 2021. In this section, we first discuss the consistency and inconsistency between the statistical results and the initial suppositions upon which the research questions were posited. Regarding results after clustering, we summarized the integration of the results and comprehensive interpretation in Table A1 in Appendix B (this is placed in the Appendix to improve readability).

First, we discuss the results of the analysis of the first part of the questions (conjoint analysis part). Samples comprising men did not show statistically significant changes (Table 2). Samples of women showed significant changes (i.e., a decrease in the significance of delivery services and an increase in the basic value of use for EC platforms). Thus, under this analytical level, we partly confirmed consistency with the supposition of the research question. However, the analysis results of the clustered dataset showed a different conclusion from that of the unclustered dataset. Table 6 demonstrates a summary of the statistically observed changes after clustering. Contrary to our initial supposition, a non-negligible proportion of clusters exhibited an increase in factors including pricing, product variety, and delivery service, and a decrease in the basic value of use for EC platforms. Only Cluster 1 of women partly supported our initial supposition described in the research question: i.e., they presented a decrease and an increase in the significance of pricing and the basic value of use for EC platforms, respectively. However, this was not true for the other clusters. Thus, our results with clustered datasets demonstrated various types of changes during the pandemic.

**Table 6:** Summary of the change observed through conjoint analysis in clustered samples.

	Statistically significant decrease (% in 2018, % in 2021)	Statistically significant increase (% in 2018, % in 2021)
Significance of better pricing	Clusters of men: NA (0%, 0%) Clusters of women: 1 and 3 (63%, 58%)	Clusters of men: 2 and 4 (36%, 36%) Clusters of women: 2 (22%, 26%)
Significance of greater variety of goods	Clusters of men: 4 (9%, 13%) Clusters of women: 2 (22%, 26%)	Clusters of men: NA (0%, 0%) Clusters of women: 4 (10%, 8%)
Significance of delivery services	Clusters of men: NA (0%, 0%) Clusters of women: 5 (6%, 8%)	Clusters of men: 3 (17%, 19%) Clusters of women: 2 and 3 (44%, 37%)
Basic value of use for EC platforms	Clusters of men: 3 and 4 (26%, 32%) Clusters of women: 3 and 4 (32%, 19%)	Clusters of men: 1 (47%, 45%) Clusters of women: 1 (41%, 47%)

Note: In the cells regarding the significance of delivery services, we included clusters showing statistical significance for either fewer delivery delays, faster delivery, or both. Here, the results of women's change in significance on faster delivery might be inconsistent with the overall results (Table 2). We believe this is a result of the following factors. While the proportion of clusters showing a decrease in the perceived importance of delivery services (i.e., Clusters 1, 3, and 5) was larger than that of the opposite clusters, that change was not large except for Cluster 5. Therefore, the *p-*value of the analysis for the whole dataset showed significance due to the large sample size, while that with the clustered dataset with a smaller dataset did not show significant differences.

Second, we discuss the results of the analysis on the second part of the questions (comprehensive questions). In Table 3, we observed some statistically significant changes considered to be attributable to the pandemic, including an increase in package drops and increase in available times for receiving delivered products. Additionally, our results with the clustered dataset presented a greater variety of changes. Table 7 summarizes changes in comprehensive question items in clustered samples into three types: (a) commonly observed changes in most clusters of both men and women (i.e., an increase in the use of package drop); (b) changes similarly observed in both men and women, but showing differences due to the characteristics of consumer clusters (i.e., increases in the purchase of consumer electronics, paid membership of Amazon Prime, and receivable times); and (c) changes observed in a few consumer clusters in men or women (e.g., in dissatisfaction factors on EC platforms). Thus, the commonly observed change in most clusters of both men and women was only the increased use of package drops. The occurrence of changes in the other items depended on the clusters’ characteristics.

**Table 7:** Summary of the changes about comprehensive question items in clustered samples.

	Details
(a) Commonly observed change for most clusters for both men and women	∗ Increased use of package drop
(b) Changes that were similarly observed in both men and women but the characteristics of the consumer clusters showing it were different	∗ Increased purchases of consumer electronics ∗ Increase in paid membership for Amazon Prime ∗ Increase in receivable times
(c) Changes observed in either or a few consumer clusters for men or women	[Only men] ∗ Increase in the number of freelancers ∗ Decrease in receive by self at workplace ∗ Increase of purchases of medicine or cosmetics [Only women] ∗ Increase in age ∗ Decrease in the number of housewives ∗ Increase in the number of part-time workers ∗ Decrease in receive by self at home ∗ Increase in dissatisfaction for high costs of goods on the platform ∗ Decrease in dissatisfaction for delivery delays

In our results, we found that the change in consumers’ sense of EC platforms by the pandemic was more complex than we anticipated. This could be because the pandemic caused various changes in the needs mechanism of consumers for the platforms. We believe this is a new finding with some novelty. On these findings, we discuss the theoretical and practical implications of this study in the following sections.

### 5.1 Theoretical implications

In contrast to the initial supposition, our results show that the change in significance of factors including pricing, product variety, delivery services, and perceived basic value of platform use, presented a variety of patterns in each clustered group. Based on these results, we discuss the theoretical implications of the results of this study for each factor, including pricing, variety of goods, and delivery services.

First, on the change in consumers' sense in “pricing,” we observed a statistically significant increase in paid membership of Amazon Prime for some clusters of both men and women. Regarding platform pricing, one study suggests that pricing can be set depending on the demand elasticity among the sides according to the Ramsey rule (Rochet and Tirole, 2003). Another study indicated that the pricing scheme that fits the market naturally dominates competitors (Inoue et al., 2019b). Therefore, we propose that the pandemic decreased the degree of demand elasticity for some types of consumers and naturally allowed acceptance of paid membership. Here, notably, we demonstrated that the patterns of estimated logistic models accompanying an increase in Amazon Prime memberships were different between men and women. Its cluster of men (Cluster 1 for men) had a characteristic focus on better pricing. In our regression model, while the degree of sense of pricing did not decrease (i.e., the increase showed marginal significance), the increase in basic value ofplatform use was significant. Therefore, the increase in the value of platform use during the pandemic could absorb the cost of paid membership while sustaining consumers’ sense of better pricing. Meanwhile, the corresponding cluster of women (Cluster 2) showed characteristics of a higher focus on better pricing and delivery services. These characteristics were largely changed compared to those in 2018, while the increase in focus on better pricing and faster delivery was significant. However, this cluster also exhibited a significant increase in dissatisfaction owing to the high costs on the platform. For this cluster, the increase in focus on faster delivery during the pandemic could absorb the cost of paid membership. Thus, we found an increase in paid membership, especially in Amazon Prime, during the pandemic, and different paths for achieving it in men and women. We consider that these findings introduce the new aspect of research on the pricing of EC platforms.

Second, on changes in consumers' sense of a “variety of goods,” we confirmed that some clusters showing a high sense of a greater variety of goods weakened their features. This was observed in Cluster 4 in men and in Cluster 2 in women. Although Cluster 4 of women exhibited an increase in the sense of a variety of goods instead of Cluster 2, its samples were less than half that of Cluster 2. As in Section 2, the variety of products can be considered a basic factor in the indirect network effect. Therefore, the weakening of a sense of a variety of goods by certain consumer groups means a decrease in the strength of indirect network effects on the consumer side for those groups. In this sense, we considered that the acceleration for the use of EC platforms might make certain consumer groups consider a high degree of product variety on the platforms natural for EC platform businesses. Thus, this study presented a change in consumers’ sense of good variety since the pandemic. This could be related to a new aspect of the research on a variety of goods on EC platforms, which was justified by the theory of the long tail (Brynjolfsson et al., 2011).

Third, regarding the change in consumers' sense in “delivery services,” we found that the most evident change was in the use of package drops. This change was commonly observed in most clusters, showing either statistical significance or marginal significance. Stable and high-quality delivery in EC platforms is significant for the evolution and persistence of platform-based markets (Inoue et al., 2019) and is related to consumer satisfaction (Chen et al., 2011; Vasić et al., 2021). We consider that the increase in acceptance of package drops significantly contributes to these aspects. Specifically, using package drops supports logistic firms' optimal delivery scheduling by reducing the time of delivery by hand and redelivery, enhancing delivery stability and shortening delivery time. Additionally, using package drops will never result in a bad impression of face-to-face timing between the delivery person and consumers. Although package drops simplify delivery services, we did not confirm a relationship between an increase in package drops and a decrease (or even increase) of the perceived significance of delivery services on platform use. This implies that using package drops might not be connected to consumers' requirements for the delivery service level. Thus, this study demonstrated that the pandemic changed consumers’ acceptance of new types of delivery services on EC platforms. Additionally, this study explored a new direction of research on the new types of delivery services on EC platforms.

### 5.2 Practical implications

This study showed some changes in consumers’ sense of values for factors on EC platforms during the pandemic. We previously observed a weakening of the value of goods variety for consumers in some clusters. Hence, the significance of the long tail could have been decreased by the pandemic in certain consumer groups. Moreover, our results suggest a new direction for good variety on the platforms. Specifically, in some clusters of both men and women, the acceptability of the purchase of consumer electronics, which are expensive and difficult to transport, increased. Thus, platform owners could seek new business opportunities for the product type, which has become more accepted on EC platforms since the pandemic. Additionally, remarkable developments in the use of package drops have been observed. This could be caused not only by an increase in the use of EC platforms but also by efforts to prevent the spread of COVID-19. As discussed in the previous section, increased acceptance of package drops can improve delivery service stability and quality, ultimately contributing to the evolution and persistence of platform-based markets. Therefore, further development of this would be quite advantageous for platform owners and delivery firms. Thus, this study proposes some opportunities generated by the pandemic for EC platform owners.

This study's findings might be applied not only to EC platform firms but also retailers managing online sales. This study's results showed that the pandemic influenced various changes in consumers' focus on pricing, product variety, and delivery services. Therefore, consumers who have not used online retail so far might become new customers. In particular, consumers like Cluster 1 of women, which came to have a lower focus on pricing and a higher perceived value of EC platforms under the pandemic, could be candidates for such customers. Additionally, the results of the analysis of the comprehensive questions could provide some implications. For example, some clusters have shown an increase in the purchase of consumer electronics using EC platforms. This implies that some consumers may increase the purchase of products that are expensive and unwieldy using online channels as part of the pandemic lifestyle. Additionally, this study observed an increase in the acceptance of package drops. Since this style of delivery could improve cost structure in delivery, more retailers, especially of low-priced products, would be able to adopt online sales more easily. Thus, this study implies that the changes in EC consumers observed in this study would be related to expanded online sales not only for EC platforms but also retailers.

Another aspect is the increase in paid memberships of Amazon Prime. Our results showed a significant increase in paid membership of Amazon Prime, while the other two major EC platforms in Japan (Rakuten Premium and Yahoo! Premium) did not experience such a change. Considering these differences, we found that Amazon Prime had a higher level of omni-channel strategy than other platforms. For example, paid membership in Amazon Prime includes not only faster delivery services but also Prime Video (video streaming service with no extra charge), Prime Reading (free books), and so on. These additional benefits for consumers could contribute to a significant increase in Amazon Prime members on EC platforms. Thus, the results of this study indicated that platforms following an omni-channel strategy could contribute to capturing more members at accidental business opportunities (e.g., the COVID-19 pandemic). This result also implies the significance of the design of mechanisms promoting synergy among managing platforms. If no synergetic mechanisms can be found on the platforms, the platform owner would miss an opportunity in the currently changing business environment. To design these mechanisms, platform owners must understand the nature of their platform and determine the types of changes in the environment that could contribute to achieving their omni-channel strategies. Thus, this study implies that platform owners managing multiple platforms should intentionally design synergistic mechanisms among platforms to effectively seize the opportunities accompanying any change in the business environment.

### 5.3 Limitations and future directions

This study has several limitations and provides related directions for future research. First, the dataset used in this study was limited to Japanese consumers. Although our results support an interpretation of the phenomena of the changes in consumers’ behavior on general EC platforms during the pandemic, we also consider that the results could be influenced by such factors as national culture, the degree of diffusion of EC platforms before the pandemic, and the degree and length of city lockdowns. Additionally, people in different countries could have different reasons for adopting EC platforms (Nathan et al., 2019). Furthermore, while this study focused on Amazon, Rakuten, and Yahoo as major EC platforms in Japan, major platforms could differ by country and might address the COVID-19 pandemic differently. We consider that the limitation on the generalizability of the results caused by these differences would not be inevitable, as this study adopted a case study approach. Therefore, future studies investigating other nations using similar techniques as in this study might find differences in the changes during the pandemic due to such factors. However, we believe that finding different results caused by such differences among countries would be also valuable to further understand how pandemic impacted EC consumers.

Second, this study's data were captured under pandemic conditions. Focusing on the diffusion of EC platforms during the pandemic in the diffusion of innovation by the crisis (Dannenberg et al., 2020), this study has room for additional investigation after the pandemic. Specifically, after this pandemic ends, the changes observed in this study could remain, reverse, or possibly advance. Thus, future studies could investigate changes in consumer behavior on EC platforms after the pandemic.

Third, this study mainly focused on the pandemic's positive aspects as further diffusion of EC platforms. However, some studies have suggested that some negative aspects of EC platforms are also caused by the pandemic. For example, some studies have reported that an increase in online shipping during the pandemic places a burden on logistics networks and forced logistics organizations to further optimize (Li et al., 2020; Villa and Monzón, 2021; Zhang and Xu, 2021). Thus, changes in EC platforms owing to the pandemic have several aspects, and future studies could investigate such changes.

Finally, the analytical object of this study was limited to only one type of EC platform defined as “platforms physically delivering various goods for consumers” such as Amazon (i.e., the general EC platform). Therefore, our results may not apply to other platforms. For example, platforms focusing on a single type of goods, such as Uber Eats, may show different patterns of change during the pandemic. Service matching platforms potentially accompanying tourism industries such as Expedia, Uber, and Airbnb may also show different changes, because such service industries were harmed by the pandemic. Additionally, if a platform's business model is different, different significant factors could occur not restricted to pricing, product variety, and delivery services. For example, factors related to user innovation could be significant in social network platforms (Qi et al., 2021). Application or software platforms would require innovations by third parties as significant factors (Inoue, 2021). Service delivery styles and customer involvement on the service development could become significant in service delivery platforms (Inoue et al., 2020). Some platforms largely use crowd workers in platforms rather than formal firms (Allen et al., 2018). Thus, future studies could focus on other types of platforms and investigate the changes in other significant factors and mechanisms in the platforms caused by the pandemic.

## 6 Conclusions

This study aimed to adduce theoretical and practical implications of the changes in consumers' EC platform use dynamics during the pandemic. We adopted an exploratory study and conducted a comparative analysis of the datasets between November 2018 and January 2021 for Japanese EC platform consumers. This study confirmed changes in consumers’ dynamics of EC platform use in three factors: pricing, variety of goods, and delivery services. Moreover, it yielded implications for both platform research stream and practitioners from comparison results, and provided directions for future work such as the further investigation of other countries, comparative analysis after the pandemic, research about other factors not focused on in this study, and investigation of other types of platforms.

## Declarations

### Author contribution statement

Yuki Inoue: Conceived and designed the experiments; Performed the experiments; Analyzed and interpreted the data; Contributed reagents, materials, analysis tools or data; Wrote the paper.

Masataka Hashimoto: Conceived and designed the experiments; Performed the experiments; Contributed reagents, materials, analysis tools or data; Wrote the paper.

### Funding statement

This work was supported by the collaborative research fund of the BCP/SCM Laboratory at Meiji University (representative of Masataka Hashimoto).

### Data availability statement

Data will be made available on request.

### Declaration of interests statement

The authors declare no conflict of interest.

### Additional information

No additional information is available for this paper.

## Figures and Tables

**Table:** 

	9 am–noon	Noon–3 pm	3 pm–6 pm	6 pm–9 pm
Monday	□	□	□	□
Tuesday	□	□	□	□
Wednesday	□	□	□	□
Thursday	□	□	□	□
Friday	□	□	□	□
Saturday	□	□	□	□
Sunday	□	□	□	□

**Table:** 

Pricing	Variety of goods	Probability of delay in delivery	Minimum delivery period	Answer
1,000 yen discount	1/4 of the store	0%	0 days	○ Use the platform ○ Use a retail store
1,000 yen discount	1/2 of the store	25%	3 days	○ Use the platform ○ Use a retail store
1,000 yen discount	Same level as the store	50%	6 days	○ Use the platform ○ Use a retail store
1,000 yen discount	Double the store	75%	9 days	○ Use the platform ○ Use a retail store
1,000 yen discount	Four times the store	100%	12 days	○ Use the platform ○ Use a retail store
500 yen discount	1/4 of the store	25%	6 days	○ Use the platform ○ Use a retail store
500 yen discount	1/2 of the store	50%	9 days	○ Use the platform ○ Use a retail store
500 yen discount	Same level as the store	75%	12 days	○ Use the platform ○ Use a retail store
500 yen discount	Double the store	100%	0 days	○ Use the platform ○ Use a retail store
500 yen discount	Four times the store	0%	3 days	○ Use the platform ○ Use a retail store
Same as the store	1/4 of the store	50%	12 days	○ Use the platform ○ Use a retail store
Same as the store	1/2 of the store	75%	0 days	○ Use the platform ○ Use a retail store
Same as the store	Same level as the store	100%	3 days	○ Use the platform ○ Use a retail store
Same as the store	Double the store	0%	6 days	○ Use the platform ○ Use a retail store
Same as the store	Four times the store	25%	9 days	○ Use the platform ○ Use a retail store
500 yen more expensive	1/4 of the store	75%	3 days	○ Use the platform ○ Use a retail store
500 yen more expensive	1/2 of the store	100%	6 days	○ Use the platform ○ Use a retail store
500 yen more expensive	Same level as the store	0%	9 days	○ Use the platform ○ Use a retail store
500 yen more expensive	Double the store	25%	12 days	○ Use the platform ○ Use a retail store
500 yen more expensive	Four times the store	50%	0 days	○ Use the platform ○ Use a retail store
1,000 yen more expensive	1/4 of the store	100%	9 days	○ Use the platform ○ Use a retail store
1,000 yen more expensive	1/2 of the store	0%	12 days	○ Use the platform ○ Use a retail store
1,000 yen more expensive	Same level as the store	25%	0 days	○ Use the platform ○ Use a retail store
1,000 yen more expensive	Double the store	50%	3 days	○ Use the platform ○ Use a retail store
1,000 yen more expensive	Four times the store	75%	6 days	○ Use the platform ○ Use a retail store

**Table A1:** Integration of the results and comprehensive interpretation.

**(a) Results for men**				
	Cluster 1	Cluster 2	Cluster 3	Cluster 4
Patterns of sense of value in 2018	Focusing on better pricing (47% of the samples)	Neutral (27% of the samples)	Focusing on faster delivery (17% of the samples)	Focusing on greater goods variety and fewer delivery delays (9% of the samples)
Patterns of sense of value in 2021	Focusing on better pricing (45% of the samples)	Neutral (23% of samples)	Focusing on faster delivery (19% of the samples)	Focusing on fewer delivery delays (13% of the samples)
Characteristic change in patterns of sense of value	Increase in basic value on using EC platform	Increased focus on better pricing	An increase in focus on faster delivery	Increased focus on better pricing and decreased focus on greater goods variety
Characteristic change in various items on EC platforms	∗ Increased use of package drop ∗ Increase in freelancers ∗ Increase of paid membership for Amazon Prime	∗ Increased use of package drop	∗ Increased use of package drop ∗ Increased purchase of consumer electronics	∗ Increased use of package drop ∗ Increase in receivable times ∗ Decrease in receivable by self at workplace ∗ Increased purchased of medicine or cosmetics
Comprehensive interpretation of results (excluding increase in use of package drop, which was commonly observed)	Users in this cluster expect lower costs for products purchased on EC platforms and is the largest group of EC users. During the pandemic, the basic value on using platforms of this group increased, while acceptability for paid membership (especially, Amazon Prime) increased against the characteristic of this cluster.	Users in this cluster were neutral for the four major factors in platform use. During the pandemic, except for an increased sense on pricing, little change in behavior was found.	Users in this cluster expect faster delivery on EC platforms. During the pandemic, this characteristic was further strengthened. Moreover, acceptability of purchases of consumer electronics, which are expensive and difficult to transport, increased on EC platforms.	Users in this cluster expected greater goods variety and stability of delivery on EC platforms. During the pandemic, expectation for greater goods variety was weakened, while that for cheaper goods increased. Additionally, receivability at home increased.

**(b) Results for women**					
	Cluster 1	Cluster 2	Cluster 3	Cluster 4	Cluster 5
Patterns of sense of value in 2018	Focus on better pricing (41% of the samples)	Focus on greater goods variety (and relatively focus on better pricing in comparison with Cluster 5) (22% of the samples)	Focus on faster delivery (22% of the samples)	Focus on fewer delivery delays (10% of the samples)	Focus on greater goods variety (and relatively focus on better delivery services in comparison with Cluster 2) (6% of the samples)
Patterns of sense of value in 2021	Focus on better pricing (47% of the samples)	Neutral (26% of the samples)	Focus on faster delivery and fewer delays in delivery (11% of the samples)	Focus on greater goods variety and fewer delivery delays (8% of the samples)	Focus on greater goods variety (8% of the samples)
Characteristic change in patterns of sense of value	Decreased focus on better pricing, while increase in basic value on using EC platform	Decreased focus on goods variety, while increased focus on faster delivery	Decreased focus on better pricing and basic value on using EC platform, while increased focus on fewer delivery delays	Increased focus on greater goods variety, while decrease of basic value on using EC platform	Decreased focus on faster delivery
Characteristic change in various items on EC platforms	∗ Increased use of package drop ∗ Increase in age ∗ Decrease in housewives, and increase in part-time workers ∗ Increase in receivable times ∗ Decrease in receive by self at home ∗ Increased purchase of consumer electronics ∗ Decrease in dissatisfaction for delivery delays	∗ Increased use of package drop ∗ Increase in receivable times ∗ Decrease in receivability in person at home ∗ Increased dissatisfaction due to high costs on the platform ∗ Increased paid membership for Amazon Prime	∗ Increased use of package drop	No statistical significance	No statistical significance (increased use of package drop showed marginal significance)
Comprehensive interpretation of results (excluding increase of use of package drop which were commonly observed)	Users in this cluster expect lower costs for products purchased on EC platforms and comprise the largest group in EC users. During the pandemic, this characteristic was weakened. The proportion of workers increased, customer segment expanded, receivability in person decreased, and purchase of consumer electronics which are expensive and difficult to transport increased on EC platforms.	Users in this cluster expected greater goods variety. During the pandemic, this characteristic decreased. Rather, this cluster became focusing other factors.	Users in this cluster expect faster delivery. During the pandemic, they further expected stability of delivery.	Users in this cluster expected fewer delivery delays. During the pandemic, they additionally focused on greater goods variety.	Users in this cluster expected greater goods variety. During the pandemic, they further deprioritized faster delivery.

## Appendix A

We present our questionnaire in this appendix. Although the original questionnaire was written in Japanese, we provide the translated version. The question items that were not used in this study are shown in (†). They were not used because they either did not match with the purpose of this study, have similar meaning with other questions (removal of duplication), or were not statistically significant (removal to improve clarity).

● Screening questions

Explanation:

In this survey, we ask you about platforms characterized as “websites or applications that allow you to purchase various products via the Internet and arrange delivery of these products to any destinations,” such as Amazon.com, Rakuten Ichiba, or Yahoo! Shopping.

∗ In this survey, we only ask you about platforms that are able to deliver purchased products.
∗ If you use multiple platforms, please provide the overall condition of the platform used.
∗ Please answer about situation of personal use (please do not include situations for business purposes).
∗ Please answer about the condition of the platform used in the past six months.

SQ 1. How frequently do you use these platforms? Please select the most appropriate answer.

○ Every day; ○ Four–five times a week; ○ Two–three times a week; ○ Once a week; ○ Two–three times a month; ○ Once a month; ○ Once every 2–3 months; ○ Once in 4–6 months; ○ Less often

SQ 2. What types of products do you purchase on these platforms? Please select all appropriate answers:

□ products for personal use; □ daily goods; □ non-daily goods; □ goods for special days

● Main questions († means question items that were not used in this study)

Explanation (same with the screening part):

In this survey, we ask you about platforms characterized as “websites or applications that allow you to purchase various products via the Internet and arrange delivery of these products to any destinations,” such as Amazon.com, Rakuten Ichiba, or Yahoo! Shopping.

∗ In this survey, we only ask you about platforms that are able to deliver purchased products.
∗ If you use multiple platforms, please provide the overall condition of the platform used.
∗ Please answer about situation of personal use (please do not include situations for business purposes).
∗ Please answer about the condition of the platform used in the past six months.

MQ 1. Please select all frequently purchased goods on the platforms.

□ Book or journal; □ DVD, music CD, or video game hardware/software; □ Consumer electronics; □ Computer-related supplies; □ Household utensils; □ Food or drink; □ Medicine or cosmetics; □ Children products or toys; □ Clothing or accessories; □ Sporting or outdoor; □ Motor vehicle related; □ Industrial machinery or R&D related; □ Others

MQ 2 (†). Please indicate the average amount paid per purchase on the platform.

(JPY)

MQ 3 (†). Please indicate your most frequently used platforms.

( )

MQ 4 (†). Regarding the probability of appointing a delivery date and time at use of the platforms, please select the most appropriate answer.

○ 0–20%; ○ 21–40%; ○ 41–60%; ○ 61–80%; ○ 81–100%

MQ 5 (†). Regarding the probability of asking for redelivery, please select the most appropriate answer.

○ 0–20%; ○ 21–40%; ○ 41–60%; ○ 61–80%; ○ 81–100%

MQ 6. Please select all available times to receive delivered products.

MQ 7. Please write percentage for use of each way of receiving goods.

Self at home (%); Self at workplace (%); Family or friends (%); Delivery locker (%); Collecting after using interim storage services at convenience stores or delivery firms (%); Use of package drop (also known as unattended delivery) services (%); other ways (%)

MQ 8. Please all current paid-up memberships.

□ Amazon Prime, □ Rakuten Premium, □ Yahoo! Premium, □ others, □ no paid membership.

MQ 9 (†). Please select all appropriate answers for your motivation regarding the use of platforms.

□ Cheapness of products, □ High variety of products, □ Saving effort to go to shops, □ Quickness on obtaining products, □ Delivery service, □ Purchasability of rare items, □ Others

MQ 10. Please select all frequently felt dissatisfaction factors at platform use.

□ Expensive, □ Low variety of goods, □ Excessive packaging, □ Delay in delivery, □ Rough treatment of parcel, □ Bad attitude of delivery person, □ Degradation of perishables, □ Others

MQ 11 (†). How expensive or cheap do you feel the total payment amount at platform use (including platform and delivery fees) compared with other major ways.

○ 50% cheaper, or less; ○ 40% cheap; ○ 30% cheap; ○ 20% cheap; ○ 10% cheaper; ○ same level; ○ 10% more expensive; ○ 20% more expensive; ○ 30% more expensive; ○ 40% more expensive; ○ 50% more expensive, or more

MQ 12 (†). How many or less do you feel the product variety of platforms compared with other major ways?

○ 0.25 times, or less; ○ 0.5 times; ○ 0.75 times; ○ same level; ○ 1.25 times; ○ 1.5 times; ○ 1.75 times; ○ 2 times; ○ 2.5 times; ○ 3 times; ○ 5 times; ○ 10 times; ○ 20 times; ○ 50 times, or more.

MQ 13 (†). Please write the probability that delivery is accomplished on time on your platform use.

(%)

MQ 14 (†). Please write the average delivery time for your platform use.

(days)

MQ 15 (†). Please select all parts that became the cause of the delay in delivery on platform use.

□ Platform firms (e.g., Amazon, Rakuten, and Yahoo), □ Exhibitors/sellers, □ Delivery firms, □ Others, □ Non-experience of delay in delivery

MQ 16. Please imagine a situation where you buy products priced in a general retail store as 5,000 Japanese yen (about USD 45–50). If following conditions about a platform are proposed to you, will you use the platform? Alternatively, will you use a retail store? Here, please define these situations accordingly: (a) amount of payment includes platform fee and delivery fee, (b) the way of receiving goods is self at home, and (c) any other factors will not influence on the platform use.

(Proposed 25 patterns in random order)

## Appendix B

Table A1 summarizes the analytical results shown in Tables 4 and 5. The summary is categorized by men and women and describes the comparison results between 2018 and 2021 for each cluster.
